# S1 is associated with chronic low back pain: a functional and structural MRI study

**DOI:** 10.1186/1744-8069-9-43

**Published:** 2013-08-21

**Authors:** Jian Kong, Rosa B Spaeth, Hsiao-Ying Wey, Alexandra Cheetham, Amanda H Cook, Karin Jensen, Ying Tan, Hesheng Liu, Danhong Wang, Marco L Loggia, Vitaly Napadow, Jordan W Smoller, Ajay D Wasan, Randy L Gollub

**Affiliations:** 1 Department of Psychiatry, Massachusetts General Hospital, Charlestown, Harvard Medical School, 120 2nd Ave., Room 101, Charlestown, MA 02129, USA; 2 A.A. Martinos Center for Biomedical Imaging, Massachusetts General Hospital, Charlestown, MA USA; 3 Departments of Anesthesiology and Psychiatry, Brigham and Women’s Hospital, Harvard Medical School, Chestnut Hill, MA USA

**Keywords:** Chronic low back pain, fMRI, Functional connectivity, Cortical thickness, Primary somatosensory cortex

## Abstract

A fundamental characteristic of neural circuits is the capacity for plasticity in response to experience. Neural plasticity is associated with the development of chronic pain disorders. In this study, we investigated 1) brain resting state functional connectivity (FC) differences between patients with chronic low back pain (cLBP) and matched healthy controls (HC); 2) FC differences within the cLBP patients as they experienced different levels of endogenous low back pain evoked by exercise maneuvers, and 3) morphometric differences between cLBP patients and matched HC. We found the dynamic character of FC in the primary somatosensory cortex (S1) in cLBP patients, i.e., S1 FC decreased when the patients experienced low intensity LBP as compared with matched healthy controls, and FC at S1 increased when cLBP patients experienced high intensity LBP as compared with the low intensity condition. In addition, we also found increased cortical thickness in the bilateral S1 somatotopically associated with the lower back in cLBP patients as compared to healthy controls. Our results provide evidence of structural plasticity co-localized with areas exhibiting FC changes in S1 in cLBP patients.

## Introduction

Chronic low back pain (cLBP) is one of the most common reasons for all physician visits in the USA and is a leading cause of disability [1,2]. The etiology of cLBP is heterogeneous [3]; non-specific cLBP, which is characterized by a lack of recognizable pathology, represents the majority of cLBP patients [3-5]. The financial cost associated with LBP care is staggering; however, treatment for cLBP has achieved limited success [6]. To develop more effective treatments, it is crucial to understand the underlying neurobiology of cLBP.

Recent neuroimaging studies [7-11] have found significant differences in brain functions when comparing patients with cLBP and matched healthy controls (HC). Compared to HC, cLBP patients showed augmented activation in pain related brain regions [9,12] during administration of comparable experimental pain, differences in activation during emotional decision-making tasks [13], reduced deactivation in several key default mode network (DMN) brain regions during a simple visual attention task [14], and increased high-frequency BOLD oscillations circumscribed mainly to the mPFC and parts of the DMN [8]. More recently, using arterial spin labeling, our group has found [11] that provoked increases in endogenous LBP ratings were positively associated with statistically significant increases in regional cerebral blood flow in a widespread network of cortical areas, including the bilateral medial and dorsolateral prefrontal cortices (mPFC and DLPFC), superior parietal lobules, S1 and S2, and unilaterally in the right insula in cLBP patients. In addition, we [15] also found that compared with healthy controls, patients demonstrated stronger default mode network connectivity to the pregenual anterior cingulate cortex, left inferior parietal lobule, and right insula. Patients' baseline chronic low back pain intensity was significantly correlated positively with connectivity strength between the DMN and right insula. Taken together, results from previous studies indicate that chronic low back pain is associated with both sensory and overall changes in brain function.

In addition to changes in brain function, multiple morphometric imaging studies report structural changes in the brains of patients with cLBP [16-21]. For instance, using voxel based morphometry (VBM) methods, Apkarian and colleagues [16] found that cLBP patients showed 5-11% less neocortical gray matter volume than control subjects and that the decreased volume was related to pain duration. These studies indicate that cLBP is also associated with structural pathological changes in the brain.

Few studies have been performed to simultaneously investigate the functional and structural changes in a single study. In one of the few such studies, Seminowicz and colleagues [22] found that cLBP is associated with decreased cortical thickness and abnormal activity during attention-demanding tasks in the DLPFC. Most intriguingly, after successful treatment, both cortical thickness and functional connectivity (FC) of the DLPFC were normalized. In another study, Baliki and colleagues [23] found that when low back pain persisted for one year, the gray matter density decreased significantly in brain regions including the striatum and insula, and left sensorimotor cortex. Additionally, they found that increased FC of the nucleus accumbens with the medial prefrontal cortex during the subacute stage of back pain predicted pain persistence. In one study from our group on fibromyalgia (FM) [24], we found that FM patients displayed a distinct overlap between decreased cortical thickness, brain volumes and measures of functional regional coherence in the rostral anterior cingulate cortex. The morphometric changes were more pronounced with longer exposure to FM pain.

In this study, we investigated 1) brain resting state FC differences between cLBP patients and matched HC; 2) FC differences within the cLBP patients as they experienced different levels of endogenous low back pain (before and after exercise maneuvers), and 3) morphometric differences between cLBP patients and matched HC. There are multiple methods to perform FC; here, we applied a whole-brain voxel-by-voxel hub FC method described in previous studies [25,26]. The basic analytic strategy was to compute an estimate of the FC of each voxel within the brain by performing Pearson correlation between each voxel and all other voxels of the whole brain [25]. One advantage of this method, we believe, is that rather than focusing on the FC between different brain regions/networks, it focuses on the synchrony of each brain region, which could facilitate the identification of the key regions disrupted in cLBP.

We believe that a combined investigation of both structure and FC changes between cLBP patients and HCs, as well as the dynamic change of FC when cLBP patients were experiencing different endogenous low back pain intensities, will shed new light on our understanding of pathophysiological mechanism, adaptation and reorganization of brain structure and function in chronic pain conditions.

## Materials and methods

### Participants

18 cLBP patients (age = 36.1 ± 9.9, 6 males) and 18 healthy controls, matched for age and gender, completed the study. The Institutional Review Board at Massachusetts General Hospital approved the study and all subjects gave written informed consent.

All patients were clinically diagnosed with nonspecific cLBP with a duration of at least six months by a clinical evaluation, including the use of X-ray/MRI reports, when available. Only those patients meeting Quebec Low Back Pain Task Force classification criteria for Classes I or II were enrolled [27]. For instance, patients reporting radicular pain and/or numbness below the knee were excluded. Subjects were also excluded if they reported major systemic diseases, or history of head injury or coma. cLBP patients were asked to rate their pain using a visual analog scale (0 no pain, 10 maximum imaginable pain). Depressive symptoms were assessed using the Beck Depression Inventory (BDI-II) for all patients who participated in the study [28]. All questionnaires were administered immediately prior to brain scanning. Demographics, clinical assessments and characteristics for cLBP patients and HCs are presented in Table 1. Healthy controls, matched on gender, age and race, were recruited through the flyers/emails in the community. All subjects passed the health subjects screening procedures, indicating that they did not have low back pain.

**Table 1:** Demographics and clinical characteristics for cLBP patients and controls

**Patients**							**Controls**		
**ID**	**Gender**	**Age**	**Race**	**BDI**	**Duration (yrs)**	**BPI (avg)**	**Gender**	**Age**	**Race**
1	F	48	White	13	3	7	F	47	White
2	M	41	Asian	8	4	6	M	37	Asian
3	F	49	Black	30	8	6	F	50	Black
4	F	47	Hisp.	7	3	10	F	49	Black
5	F	23	White	1	10	3	F	26	White
6	M	27	White	0	10	3	M	30	White
7	F	23	White	4	3	3	F	23	White
8	M	38	White	0	2	4	M	39	White
9	M	25	Multi.	0	5	3	M	27	White
10	F	44	White	9	12	4	F	45	White
11	M	30	Multi.	5	10	9	M	34	White
12	F	31	Black	1	2	6	F	32	Black
13	F	47	Black	3	5	8	F	47	Black
14	F	46	Black	9	3	6	F	46	Black
15	F	46	White	8	10	5	F	47	White
16	F	34	Black	10	3	8	F	34	White
17	M	26	White	0	1.5	2	M	27	White
18	F	25	Asian	9	0.5	2	F	28	Asian
**Avg**	**12 F**	**36.11**		**6.5**	**5.28**	**5.28**	**12 F**	**37.11**	

### Medication

Medication use per self-report was limited to non-steroidal anti-inflammatory drugs (NSAIDs, e.g., ibuprofen, Motrin, Advil, and Naproxen) and acetaminophen (e.g., Tylenol). Additional non-pharmacological methods of self-reported pain management included chiropractic massages, physical therapy, exercises, and acupuncture.

### MRI data acquisition and pain exacerbation procedures

All MRI data were acquired with a 3T Siemens whole-body scanner with echo-planar imaging capability using a 32-channel radio-frequency head coil at the Martinos Center for Biomedical Imaging. During the resting state fMRI scan, subjects were asked to keep their eyes open and look at a darkened screen for 6 minutes. The BOLD fMRI scan acquisition included 47 slices with slice thickness of 3 mm, TR 3000 ms, TE 30 ms, and a 3×3 mm in-plane spatial resolution. T1-weighted MPRAGE type structural images were acquired using the following parameters: voxel size 1.2×1.2×1.2 mm, TR, 2.2 s, TE, 1.54 ms, flip angle: 7 degrees, slices: 144; field of view: 230. Before and immediately after each 6 minute scan, subjects were asked to rate the intensity of their LBP using the 0–10 pain scale.

After the first resting state scan, cLBP patients were taken out of the scanner to perform exercises for a period lasting up to 10 minutes to exacerbate their endogenous lower back pain. These exercises were tailored to each patient based on their report of which movements exacerbated their pain. They included lumbar flexion and extension exercises, sit-ups, or lumbar rotation exercises, where the subject rotated his or her body from side to side at a self-selected speed. The patients were required to perform their exercise(s) slowly, and at maximum flexion/extension, such that they might feel the exercise trigger low back pain. During the screening, all subjects were asked to confirm that they could perform these exercises. If at the end of the first resting state scan the patient’s cLBP pain rating was too strong (≥ 7 in 0–10 scale) and the patient was reluctant to perform exercises to enhance their pain experience, they were asked to wait for 10 minutes in a comfortable position that they chose before starting the second half of the scanning session.

During the exercises, cLBP patients were asked intermittently to report their level of pain using the 0–10 pain scale; the exercises were repeated until subjects reported an increase in pain of approximately 3 points on the pain scale. Once this level of pain was achieved, subjects were placed back in the scanner to repeat the same MRI scans that were acquired before the exercise maneuvers. For healthy controls, structural and resting state scans were only collected once.

### Whole brain voxel-wise functional connectivity analysis

To summarize the procedures, data pre-processing included deleting the first four volumes to remove non steady-state images, slice-timing correction, motion correction, and co-registration to the standard brain atlas (MNI152). In addition, the images were corrected for linear trends over each run, low-pass filtered at 0.08 Hz and spatially smoothed with a 4 mm FWHM Gaussian kernel. Nine nuisance parameters (six motion parameters, whole brain, white matter and CSF) were also extracted. Finally, the data were down-sampled into 4 mm isotropic resolution to reduce the computational burden.

Pearson correlation coefficients were calculated between time series data of each voxel and every other voxel of the whole brain. Cross-correlation measures were weighted by the number of strongly correlated links (i.e., correlation above a threshold of r > 0.25) across the entire brain to every given voxel to determine the degree of connectivity, and a z-transformation was applied to prepare for group comparison [25,26]. The z-transformation did not affect the topography of the connectivity map, but rather normalized each individual’s connectivity map. Since global signal removal might induce artificial negative correlations [29,30], further analyses were restricted to positive correlations.

Paired and unpaired two-sample t-tests were then used for group analysis to compare FC changes within patients with different endogenous LBP intensities, and between HCs and cLBP patients respectively using FSL. The results were cluster corrected for multiple comparisons and a threshold was set at Z > 2.3 and p < 0.05 with 10 contiguous voxels and spatially smoothed with 1mm spherical kernel for display. Mean Z values over the S1 region (Z > 2.3) were also extracted from individual subjects and plotted for different groups.

To identify networks related to the change in endogenous pain intensity, the difference in subjective pain ratings before and after the maneuver (high pain – low pain) was used as a covariate of interest for regression analysis (Z > 2.3 and p < 0.05 with 10 contiguous voxels) across the whole brain.

### Brain structure analysis using FreeSurfer

Automated cortical surface reconstruction was performed on the T1 MPRAGE scans in Free Surfer version 5.1 (http://surfer.nmr.mgh.harvard.edu) using previously described methods [31-33]. Any inaccuracies in the reconstruction of white and pial surfaces of individual subjects were manually corrected before calculating cortical thickness following instructions provided by the software developers. The cortical thickness measure was computed as the distance between the pial and white matter surfaces at each point across the cortical mantle.

Group analyses were performed by resampling each subject’s data to the FreeSurfer average atlas, distributed as a part of FreeSurfer. Cortical thickness maps were smoothed using a Gaussian kernel with a FWHM of 10 mm. Vertex wise analyses of cortical thickness were performed using FreeSurfer. For both cLBP patients and HCs, a linear model of the cortical thickness was calculated at each vertex on the surface. Two-sample t-tests were used to compare the cortical thickness between cLBP patients and HCs. Based on previous structural studies [16,18,22,23], we identified three bilateral *a priori* regions of interest (ROIs), including the lateral prefrontal cortex (LPFC), primary somatosensory cortex (S1), and insula, and restricted our analysis to a mask of these ROIs. The mask was created by merging the FreeSurfer parcellation labels for the rostral and caudal middle frontal areas, insula and the top portion of the postcentral gyrus. Based on the somatosensory homunculus as defined by Penfield [34] we included only the portion of the somatosensory cortex that include the cortical representation of the lower back (the top third of S1) in our mask using a method provided by FreeSurfer. For *a priori* ROIs, a vertex wise threshold was set at p < 0.05 corrected for multiple comparisons in FreeSurfer using Monte Carlo permutations with 5000 iterations within the mask.

In addition, we also conducted a whole brain cortical thickness comparison between cLBP patients and HCs using a vertex wise threshold (p < 0.05) corrected for multiple comparisons in FreeSurfer using Monte Carlo permutations with 5000 iterations. To further explore the relationship between brain cortical thickness changes and duration of cLBP, we also applied a regression analysis to investigate the relationship between cortical thickness and the duration of LBP within cLBP patients.

The automated segmentation procedure for labeling different brain structures and extracting their volumetric measures is described in detail by Fischl et al. [32]. This procedure assigns a neuroanatomical label to each voxel in an MRI volume based on probabilistic information automatically estimated from a manually labeled training set, including both gray and white matter. This technique has been shown to be comparable in accuracy to manual labeling [35,36]. The automatic segmentations were also visually inspected for accuracy. In the present study, we focus on the volumetric difference in the top one third of primary somatosensory cortex for the following reasons:1) previous studies have suggested the involvement of S1 in both the experimental presentation of noxious stimuli and in pathological pain states such as cLBP [11,37-39]; in particular, studies have suggested the possibility of a reorganization of S1 in cLBP patients [40] in the top portion of the postcentral gyrus, and 2) FC results from the present study showed dynamic FC changes at this region (see Results section for more details).

Using the automatic labeling of brain structures described previously, we obtained volumetric measures of the entire S1 cortical region as well as specific portions of S1, divided perpendicular to its long axis. For the present analysis, we divided S1 into 3 sections as determined by the length of the long axis. A two sample *t*-test was then applied to compare the volume differences between the patients and controls in the bilateral top third of S1. As an exploratory control, we also applied the same analysis on the total S1 volume, as well as bilateral middle and bottom thirds of S1 separately.

## Results

A total of 18 cLBP patients, and age- and gender-matched HCs, completed the study.

Please see Table 1 for more details on patient/control characteristics. One patient had strong chronic pain at baseline and thus did not perform any exercises. After lying down for 10 minutes, the patient felt a reduction in low back pain. The patient received the exact same set of scan procedures before and after the 10 minute rest period, comparable to the healthy control condition. This data was included in the data analysis (see Methods for details).

In this study, the LBP intensity was recorded before and after each resting state fMRI scan. We used the average of pre- and post-scan ratings to represent the LBP intensity level for the particular resting scan. All patients, except one who did not perform the exercises, reported an increase in low back pain intensity from pre- to post-maneuver. In our data analysis, we define the scanning period during which patients’ low back pain ratings are lower as the low pain (LP) condition, and the other as the high pain (HP) condition. The average LBP intensity was 3.8 ± 2.5 for LP condition, and 6.7 ± 2.0 for HP condition. No back pain was reported for healthy controls.

### Functional connectivity analysis results

**Figure 1:**
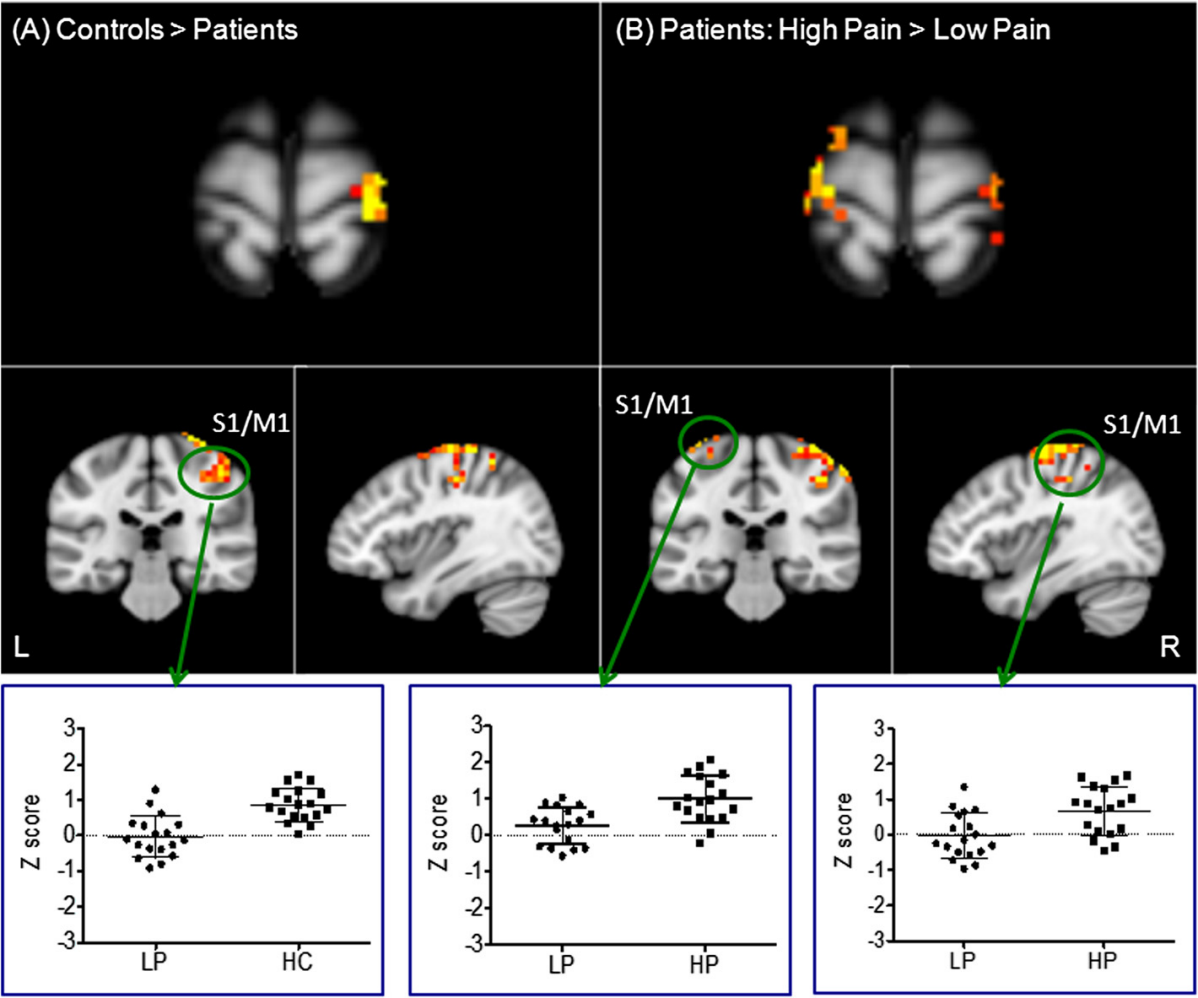
**Functional connectivity differences across different conditions. A**: Functional connectivity differences between HCs and patients with low endogenous LBP; **B**: Functional connectivity differences between the high endogenous LBP condition and low endogenous LBP condition within patients. The bar indicates the scatter plot of the representative brain region showing significant changes. The y axis indicates the average functional connectivity z value of the cluster.

Whole brain voxel-by-voxel hub FC in HCs and cLBP patients during the LP condition was compared using a two-sample *t*-test. Results showed that HCs had significantly greater FC in the right primary somatosensory and motor areas (S1 and M1) located in the upper portion of the pre- and postcentral gyrus compared with patients with cLBP LP condition (Figure 1). The opposite contrast showed that cLBP patients had greater FC in the left fusiformgyrus, occipital gyrus, right posterior cingulate cortex, and inferior parietal gyrus (Table 2). When we compared HCs with cLBP HP condition, HCs showed significant greater FC in the left superior frontal gyrus. No regions showed significant differences in the opposite comparison (Table 2).

**Table 2:** Results from whole brain voxel-by-voxel functional connectivity difference analyses among different conditions

				**Peak coordinates (MNI)**		
**Contrasts**	**Brain region**	**Cluster (mm**^**3**^**)**	**Z**_**max**_	**X**	**Y**	**Z**
High pain > low pain	R. Precentral Gyrus	7360	3.88	40	−28	66
Low pain > high pain	R. Precuneus	3928	3.5	28	−64	30
	R. Uncus	1424	3.72	28	0	−42
	R. Inferior Parietal Lobule	1088	3.68	40	−48	38
	L. Sub-gyral	1088	3.54	−36	−8	−10
Controls > low pain	R. Postcentral Gyrus	5528	3.93	44	−20	66
Low pain > controls	L. Fusiform Gyrus	2272	3.54	−44	−60	−14
	L. Precuneus	1984	3.22	−24	−72	26
	R. Inferior Parietal Lobule	1920	3.61	40	−52	38
Controls > high pain	R. Medial Frontal Gyrus	1176	3.28	4	68	10
High pain > controls	N/A					

A paired *t*-test comparing cLBP LP and cLBP HP conditions in patients showed that patients in the HP condition had greater FC in bilateral S1 and M1, and left superior frontal cortex, where as patients in the cLBP LP condition showed greater FC in the right inferior parietal lobule, cuneus, and middle occipital gyrus (Table 2 and Figure 1).

**Figure 2:**
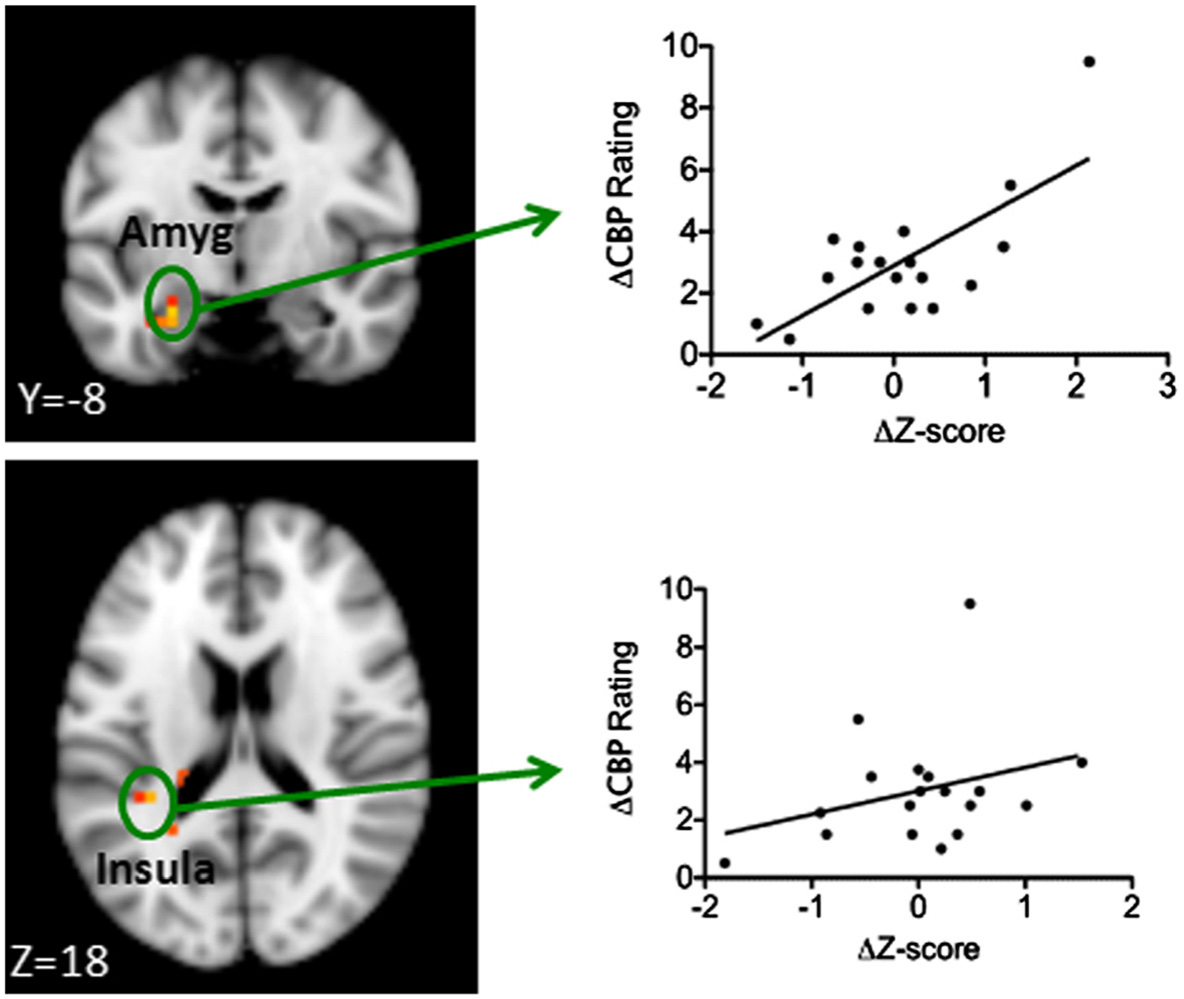
**Brain regions showing significant associations between the functional connectivity changes and corresponding LBP intensity changes.** The x-axis indicates the average functional connectivity z value changes between the high and low pain condition at representative regions, and the y-axis indicates the corresponding rating changes.

To test the association between the FC and subjective cLBP rating change, we applied a regression analysis between the FC difference of cLBP HP versus cLBP LP (HP-LP) and the corresponding low back pain rating differences. The results showed significant positive correlations between FC and LBP rating changes at the left insula, precuneus, amygdala and fusiform (Table 3 and Figure 2).

**Table 3:** Brain regions showing significant correlations between the functional connectivity difference (HP-LP) and the corresponding pain rating changes (HP-LP) in cLBP patients

			**Peak coordinates (MNI)**		
**Brain region**	**Cluster (mm**^**3**^**)**	**Z**_**max**_	**X**	**Y**	**Z**
L Insula	2176	3.8	−32	−40	22
L Precuneus	1856	3.93	−8	−68	26
L Amygdala	1152	2.86	−24	−8	−22
R Fusiform Gyrus	1088	3.23	44	−40	−18

### Structural analysis

Volumetric analysis showed that there were no significant differences between cLBP patients and controls with regard to total gray volume (p = .78), subcortical gray volume (p = 0.52) and intracranial volume (p =0.87).

**Figure 3:**
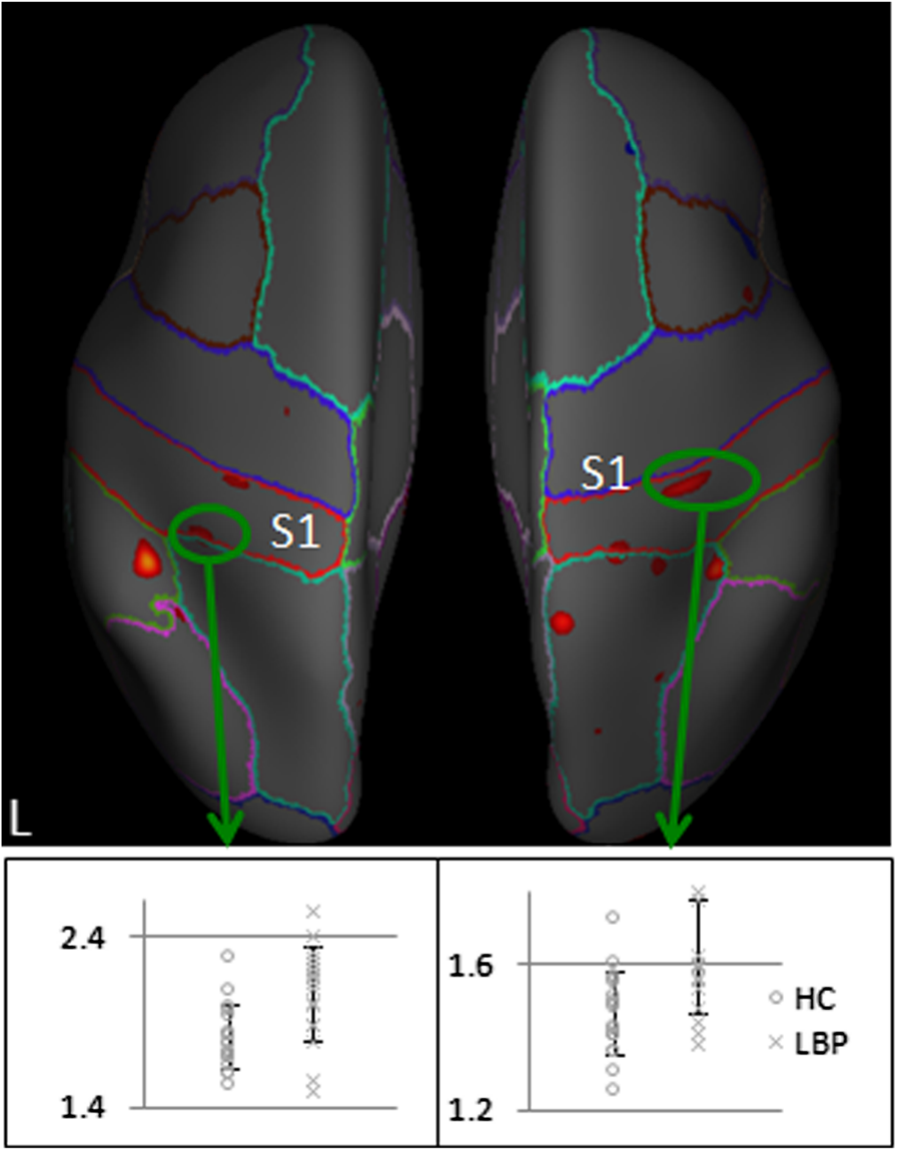
**Cortical thickness measures in LBP patients and healthy controls.** The bar indicates the scatter plot of the bilateral postcentral gyrus showed significant changes. The y-axis indicates the cortical thickness at a peak vertex in the representative region.

Direct comparison between cLBP patients and the control group showed that the cortical thickness measure of the bilateral postcentral gyri was significantly greater in cLBP patients. No other brain regions showed significant differences between the two groups (Table 4 and Figure 3).

**Table 4:** Results of the cortical thickness analysis investigating the anatomical differences between cLBP patients and healthy controls (HC)

			**Peak coordinates (MNI)**		
**Contrasts**	**Brain region**	**Cluster corrected P-Value**	**X**	**Y**	**Z**
Patients > Control	R. Postcentral Gyrus	0.001	35.1	−30.4	59.2
	L. Postcentral Gyrus	0.029	−26.8	−30.3	54.0
Control > Patients	no regions survived				

To further explore the relationship between S1 morphometric changes and cLBP, we compared the volume of the top third of the postcentral gyrus bilaterally. The results showed a significant difference in volume between the cLBP patients (mean ± SD, 5416 ± 932 mm^3^) and matched HCs (4781 ± 783 mm^3^) (LBP patients > HC) bilaterally in the top third of the postcentral gyrus (p = 0.033). When age and gender were included in the model as covariates, the p value remained significant (p = 0.17).

In a further test of volumetric differences, we compared the whole volume, middle and bottom third of the postcentral gyrus bilaterally. The results showed there were no significant differences between the cLBP patients and controls in total volume (16977 ± 2868 mm^3^vs 16198 ± 2739 mm^3^, p = .37), middle third (5696 ± 946 mm^3^ vs 5554 ± 1080 mm^3^, p = .411) and bottom third of S1 (5863 ± 1029 mm^3^ vs 5863 ± 1198 mm^3^, p = 1.0), which highlights the specificity of the changes in the top third of S1 to cLBP patients.

Voxelwise whole brain regression analysis between the cortical thickness and the duration of LBP within cLBP patients no brain regions survived the threshold we set.

## Discussion

In this study, we investigated the FC and structural difference between cLBP patients and matched HCs. The results of this study identify not only that FC differs between cLBP patients and healthy controls at S1 (the area corresponding to the lower back), but also that FC varies with endogenous low back pain intensity (high intensity pain condition showed stronger FC). In addition, we also found differences in brain structure (cortical thickness and volume) between the cLBP patients and HCs at S1. The combined neuroanatomical and functional changes of S1 imply that this brain region in particular may play an important role in the pathophysiology of cLBP.

Recently, the spontaneous brain activity fluctuations observed in resting state fMRI, have drawn the attention of brain imaging investigators [41-43]. It is believed that low-frequency components of the spontaneous functional MR imaging signal can provide information about the intrinsic functional and anatomical organization of the brain [44]. In this study, we applied a data driven method [25,26] that calculates the connectivity of each voxel with other voxels of the whole brain. The advantage of this method is that it can elucidate key regions (rather than networks) that show FC changes among different conditions. In this study, we found that the HCs demonstrated significantly greater connectivity bilaterally at S1 than cLBP patients during the LP condition (less severe endogenous low back pain). More interestingly, we found that this decreased FC at S1 during the LP condition increased as endogenous low back pain intensity increased, suggesting the dynamic character of FC at S1. The negative correlation between FC and LBP rating changes at S1 indicates that the rate of increase in FC is more dramatic when pain intensity starts to increase.

The involvement of S1 in pain processing has been known for a long time. Brain imaging studies [11,37-39,45-47] have found that S1 is activated during the presentation of noxious stimuli as well as in association with pathological pain states such as chronic low back pain. Although arguable, some investigators believe that S1 plays a prominent and highly modulated role in the sensory aspects of pain, including the localization and discrimination of pain intensity [37]. Thus, the dynamic FC changes observed at S1, in association with the varying endogenous pain intensity levels observed in our study, may represent the important role that S1 plays in monitoring different pain intensities.

In addition to FC changes, we also found increased cortical thickness in the superior section of S1 in the cLBP patients. A further analysis of brain volume in the top one third of the postcentral gyrus (S1), but not the whole S1 volume, also demonstrated that S1 was significantly larger in cLBP patients compared to HCs. This result is consistent with a recent study [48] in which the authors found increased brain gray matter(GM) density in chronic low back pain patients using a relative large cohort of patients (47 cLBP patients). It is noted that the location of the significant FC changes and cortical thickness differences in S1 is also similar to the recent study [48].

These results are also consistent with previous animal studies that suggest S1 is highly plastic during both development and in adulthood [49]. This plasticity/reorganization can occur in various situations including peripheral lesions and passive sensory experiences, including chronic pain [21,40,49,50]. In addition, our results are also in line with previous morphometric studies [51-53] that found exercise, learning and stimulation can change brain structures that are associated with the task. For instance, Teutsch and colleagues [46] found that after 8 consecutive days of identical 20-minute pain sequences, repeated painful stimulation resulted in stimulation dependent substantial increases of gray matter in S1 in healthy subjects (average age 26).

In a recent study of chronic pain (migraine) patients [54], investigators found that patients had greater cortical thickness at S1 compared to the control group. In another study [55], investigators found a significant increase in the activation response in areas of the ventral postcentral gyrus (POG) in stroke patients relative to controls. The same ventral POG areas showed a significant increase in cortical thickness in the patients.

Gray matter differences could result from changes in cell volume, synaptic densities, blood flow, or interstitial fluid [45,56]. In a recent study on healthy subjects (average age 25) [45], Erpelding and colleagues found strong correlation between greater thermal and pain sensitivity and cortical thickness of the primary somatosensory cortex, indicating that individuals who are highly sensitive to pain have thicker cortical gray matter. Studies [57-60] also suggest that cLBP patients exhibit hypersensitivity in the central nervous system as indicated by lower perception thresholds, lower pain thresholds, lower pain tolerance values, and reduced habituation compared to healthy controls. Thus, increased cortical thickness and increased volume (top one third) in the primary somatosensory cortex in cLBP patients is in line with the central sensitization of chronic pain patients and may represent some level of compensation for the constant experience of pain. One question that remains to be answered is whether the increased changes observed in the primary somatosensory cortex are the consequence of cLBP or a cause in the development of cLBP. Theoretically, individuals with thicker/larger somatosensory cortices may be more sensitive to pain, and thus be more vulnerable to developing cLBP [47]. A study including pre-pain brain structure measurements would be able to answer this question.

Although consistent with a recent study with large cohort of patients [48], our finding of increased cortical thickness at S1 is different from previous studies [18,19,22,23], in which the authors found a significant decrease in gray matter in the somatosensory cortex (S1). We believe this difference may arise from differences between subgroups of the cLBP population, an observation which has been documented in other chronic pain populations that show differential changes in brain structure (e.g., chronic head pain) [20]. In the previous studies [18,22,23] that found somatosensory gray matter decreases, all patients had spinal disk pathology changes; in contrast, subjects in our study were characterized as nonspecific cLBP patients, representing the majority of cLBP patients [4,5,61].

In other studies, a discogenic back pain subgroup may have injured the nerve surrounding the affected region and prevented the function/movement of the related regions, which caused the gray matter decrease in the corresponding somatosensory region [62]. In nonspecific cLBP, however, this may not be the case [61,63], as pain is not attributable to a recognizable specific pathology. For these patients, central vulnerable characteristics that have been associated with high sensitivity to pain [45], such as increased cortical thickness, may be unique to this subgroup of cLBP patients.

Another potential reason that we see conflicting observations (cortical thickening versus thinning) between studies is the age of study population. In a previous study [64], Schweinhardt and colleagues found vestibulodynia (PVD) patients had significantly higher gray matter densities in pain modulatory and stress-related areas, including the parahippocampal gyrus/hippocampus and basal ganglia when compared with healthy controls. In a subsequent study from the same group [65], the authors found that whereas older fibromyalgia patients (average age 56) had the commonly observed gray matter decreases, younger patients (average age 43) showed exclusively gray matter increases relative to age-matched controls in areas including the insula, basal ganglia, and ventrolateral prefrontal cortex, which provides further support of this hypothesis in a different chronic pain population.

In our study, the average age of cLBP patients was 36; the average ages in the previous studies were 50 in the Schmidt-Wilcke et al. study [18], 46 in the Seminowicz et al. study [22]; 48 [19] (n.b., the average age of healthy controls in this study is 39, which is lower than the patient population) and 43 in the Baliki et al. studies [23](the patients in this study have a total duration of only one year of chronic pain, which is much shorter than studies from other groups). Thus, we speculate that the cortical thickness increase observed in S1 in our study may be due to the relatively young age of the patients.

In this study, we also found significant positive correlations between FC changes and LBP rating changes at the left insula and amygdala. The insula is one of the key regions in pain processing [11,39,66,67]. The amygdala is a key region involved in emotion processes such as stress and anxiety [68,69]. Increased low back pain intensity may be associated with more anxiety and stress. Our result may indicate that the increased LBP intensity can be reflected by brain FC increases in brain regions associated with both pain and emotional processes.

There are some potential limitations in this study. We did not include medication in the model, and although it is unlikely for medication to affect our results, we cannot completely rule out the effect of medication. It is important to note, however, that we excluded all patients using opioids from this study, as a previous study [70] found that administering oral morphine daily for 1 month can cause anatomical changes in the brain.

Another potential limitation is the order effects between the high endogenous LBP and low endogenous LBP conditions. One challenge of cLBP studies is that once LBP is provoked, it is hard to control without any pharmacological intervention. In this study, we used exercise to provoke the patients’ LBP; thus, the high pain condition tended to follow the low pain condition. However, previous studies have suggested high test-retest reliability across different sessions [44,71], indicating that the significant findings observed in this study are unlikely due to order effects. Future studies are needed to address the potential confounding order effects in this study.

Finally, we did not collect second scan data for healthy controls. However, previous studies have suggested that resting state FC measurements have moderate to high reliability [72,73]. In a previous study [15], we investigated the FC change before and after leg lift maneuver, we found that in cLBP patients, the correlation between these connectivity patterns is significantly associated with the presence of ongoing clinical pain, but not in healthy controls who performed the same maneuver, indicating the specificity of FC change and the clinical pain intensity change. Thus, we believe the difference observed in cLBP patients before and after maneuver tend to be related to the low back pain changes rather than the random changes between the two scan sessions.

## Conclusions

In summary, our study showed that compared with healthy controls, cLBP patients have increased cortical thickness and increased volume in the top one third of S1, and decreased FC during low intensity LBP conditions at S1. Interestingly, we also found that the FC at S1 increases when patients experience more endogenous back pain. We believe our results provide the evidence of structural plasticity co-localized with areas exhibiting dynamic FC characteristics in S1 in cLBP patients.

## Competing interests

There is no conflict of interest to claim for all authors.

## Authors’ contributions

JK: experiment design, data collection, data analysis, manuscript preparation. RS: data collection, data analysis, manuscript preparation. HYW: data analysis, manuscript preparation. AC: data collection, data analysis, manuscript preparation. AC: data analysis, manuscript preparation. KJ: manuscript preparation. YT: data analysis. HSL: data analysis. DHW: data analysis. ML: manuscript preparation. VN: experiment design. JS: experiment design, manuscript preparation. AW: experiment design, manuscript preparation. RG: experiment design, manuscript preparation. All authors read and approved the final manuscript.
